# Comparison of Cervical Levels of Interleukins-6 and -8 in Patients with and without Cervical Intraepithelial Neoplasia

**DOI:** 10.31557/APJCP.2021.22.4.1225

**Published:** 2021-04

**Authors:** Zahra Vahedpour, Masoumeh Abedzadeh-Kalahroudi, Mojtaba Sehat, Ahmad Piroozmand, Maedeh Memar

**Affiliations:** 1 *Autoimmune Research Center, Kashan University of Medical Sciences, Kashan, Iran. *; 2 *Trauma Nursing Research center, Kashan University of Medical Sciences, Kashan, Iran. *; 3 *Kashan Trauma Research Center Head of Department of Community Medicine, Faculty of Medicine Kashan University of Medical Sciences, Kashan, Iran. *; 4 *Department of Obstetrics and Gynecology; Kashan University of Medical Sciences, Kashan, Iran. *

**Keywords:** Interleukins, cervical intraepithelial neoplasia, cervical mucus

## Abstract

**Introduction:**

Interleukins-6 and -8 are two pro-inflammatory cytokines increasing in serum and local levels under malignant conditions. There are limited evidences on the association between cervical level of these two factors and cervical intraepithelial neoplasia (CIN). So, this study aimed to explore the association between cervical levels of IL-6 and IL-8 with cervical premalignant lesions.

**Methods:**

The present case-control study was conducted on married women undergone Pap smear for routine screening in two groups as the group with CIN (n=100) and the healthy control group (n=100). Cervical secretions were collected using sterile swab and the levels of IL-8 and IL-6 were measured by enzyme-linked immunosorbent assay (ELISA). The obtained data were analyzed by SPSS software.

**Results:**

The mean cervical IL-6 level was 568.66±594.62 pg/ml in the patients with CIN and 212.7±213.9 pg/ml in the controls (P <0.001). The cervical IL-8 levels in the case and control groups were measured to be 1320.43±876.5 pg/ml and 1053.59±747.64 pg/ml, respectively (p=0.02). By modifying the confounding size effect of the age and marital duration, it was determined that cervical levels of IL-6 and IL-8 were both associated with CIN.

**Conclusion:**

Our results showed that the cervical levels of IL-6 and IL-8 are associated with CIN independent of age and marital duration.

## Introduction

In developing countries, cervical cancer still is the second most common cancer and the second leading cause of malignancy in women (Aminisani et al., 2016). Most cases with cervical cancer are in developing countries, with an estimated prevalence of 15.7 per 100,000 people per year, versus 9.9 per 100,000 people per year in developed countries (Bermedo-Carrasco and Waldner, 2016; Castillo et al., 2016). The mortality rate of cervical cancer in developing countries is significantly higher than that of developed countries, as 3.8 in developing countries and 3.3 in developed countries per 100,000 people (Jemal et al., 2011; Guerrero et al., 2016). The differences observed in the incidence and mortality rates of cervical cancer in developed and developing societies can be attributed to preventive measures, including the Human Papilloma Virus (HPV) vaccination program and Papanicolaou test (Pap smear) (Terán-Hernández et al., 2016; Taebi et al., 2019). The Pap test is the most common method of both screening and preventing the cervical cancer in different societies, in which premalignant and malignant lesions at the early stages can be well-recognized (Acharya and Grigsby, 2016). Various studies have shown that the use of Pap test and early detection of malignant lesions can increase the treatment rate of cervical cancer from 70% to 90%, which can significantly reduce the mortality caused by this disease (Adams et al., 2007; Fang et al., 2016). However, its sensitivity in different studies has been reported to be ranged from 10% to 75% and its specificity was within the range of 42% to 100% (Eftekhar et al., 2005, Keshavarzi et al., 2013;Vahedpoor et al., 2019).

Despite the fact that the Pap smear has been successful in the diagnosis of cervical cancer, some restrictions avoid the full effectiveness of this method. Accordingly, technical errors during sample preparation and dependency to the experienced staff for its interpretation are two factors that may prevent the detection of abnormal cells at early stages (Kulkarni et al., 2013; Gupta et al., 2016). Considering the importance of early and proper detection and prevention of unnecessary actions, it is important to enhance the accuracy and quality of Pap smear using operator-independent methods. 

Interleukin 6 (IL-6) is a multifunctional cytokine, most commonly known as B-cell differentiation. This cytokine has some functions such as the regulation of immune system and inflammatory response, the regulation of producing the hepatic acute-phase proteins, hematopoiesis, and bone metabolism (Kong et al., 2016). Different assumptions pointed to the potential role of this cytokine in the pathogenesis of cervical cancer, as the central mediator of female genital inflammation, since cervical cancer is typically caused by a close association of chronic inflammation due to infection with sexually transmitted agents (Wei et al., 2001).

Interleukin 8 (IL-8) is another inflammatory cytokine, which belongs to the CXC chemokines family. Accordingly, this plays an important role in activating neutrophils, eosinophils, and lymphocytes. As well, this cytokine is known as an effective angiogenic factor (Matsui et al., 2015; Shi and Wei, 2016). An increase in the secretion of IL-8 and its cell surface receptors has been observed in cancer cells (Waugh and Wilson, 2008). Limited studies have been conducted on this field. In a study performed by Tjiong et al. on 35 women with invasive cervical cancer, the mean cervical level of both interleukins in case group was significantly higher than that of the healthy population (Tjiong et al., 1999). In another study, Tjiong et al. reported that the cervical IL-6 levels were significantly higher in CIN patients group compared to control group (Tjiong et al., 1997). Naik et al., (2016) also observed the increased levels of IL-6 in cervicovaginal secretions in patients with premalignant and malignant cervical lesions.

It seems that measuring the levels of these two cytokines in the specific secretion of the cervix in detection of premalignant and malignant lesions, can be more accurate than measuring them in cervicovaginal secretions. Moreover, by measuring the cervical level of these interleukins, it is possible to more increase the accuracy of Pap smears, especially in cases that reliable results are not available. Considering that the studies in this field were mainly done on the sample taken from the cervicovaginal discharge (Tjiong et al., 1999; Naik Kumar et al., 2016), with small the sample size, in this study, we decided to conduct a research to compare the cervical levels of IL-6 and IL-8 in patients with cervical intraepithelial lesions and also in healthy women.

## Materials and Methods

*Methods*

*Study Population*

The current case-control study was conducted on the population of all women aged between 20 and 65 years old who were referred due to the results of colposcopy to the Clinic of Obstetrics and Gynecology at Naghavi Hospital of Kashan between September 2018 and August 2019. 

The sample size consisted of 100 patients with one type of CIN I-III disorder (as case group), and 100 healthy women (as the control group) who were selected by convenience sampling method.

*Inclusion and Exclusion Criteria*

The inclusion criteria were age range of 20 to 65 years old and being married. The exclusion criteria were having a history of gynecologic malignancy, history of surgery or gynecologic manipulation, history of autoimmune diseases, gynecologic active infection or its history, and history of non-gynecologic malignancies and HIV.

The case group sampling was performed conveniently among clients who were routinely screened for Pap smear and based on the results of cytological examinations, they underwent colposcopy and biopsy. The control group’s participants were selected from those who had normal Pap smear results, matched by age and marital duration, using convenient sampling method. All the eligible subjects (married women aged between 20 and 65 years old) were enrolled in the study until completing the required sample size. Demographic and clinical data including age, marital duration, history of chronic diseases, history of malignancy, and history of gynecological surgery were taken from the included patients and recorded in a checklist.

*IL-6 and IL-8 Assay*

All the patients were placed in the lithotomy position and cervical sampling was performed for them using a sterile Pap smear brush. The specimen was stretched on a glass slide and then fixed using fixation spray. All Pap smear slides were explored and interpreted by an experienced pathologist. In those patients with abnormal Pap smear results, colposcopy and biopsy were performed. The cervical secretions were also collected using a sterile Dacron polyester swab inserted within the 2 cm of cervical canal for ten seconds. The prepared swab was immediately inserted into 1 ml of buffer solution (containing 1% bovine serum albumin, 5 mM of Tris buffer plus ethylene ediaminetetra-acetic acid, 5 mM/l of phenyl methyl sulfonyl fluoride, and 0.5 trypsin-inhibitory unit aprotinin). The samples of cervical secretions were frozen at -70°C. The cervical levels of IL-6 and IL-8 were measured using enzyme-linked immune sorbent assays (ELISA) kit after de-freezing the samples. All the specimens, including cervical cytology and secretion, were analyzed in Shahid Beheshti Hospital laboratory. 

*Statistical Analysis*

The Kolmogorov–Smirnoff test showed that IL-6 and IL-8 levels had not normal distribution. Quantitative data were reported as mean and standard deviation and qualitative data as relative and absolute frequency. The obtained data were analyzed using independent t-test for comparison of the mean age and marital duration. As well, Mann-Whitney U test was applied for comparing cervical levels of IL-6 and IL-8 between the two groups. The regression analysis was performed to assess the confounder effects of age and marital duration on IL-6 and IL-8 levels. SPSS software version 16 was used for all analyses and P value < 0.05 was considered as a statistically significance level.

*Ethical approval*

This study was approved by the Ethics Committee of Kashan University of Medical Sciences, Kashan, Iran (Ethical code: IR.kaums.mednt.rec.1396.116 date of Issue: 2018.03.05). All the participants were informed about the objectives of the study, the confidentiality of the data, and voluntarily participation in the study. Notably, written informed consent was obtained from all the subjects.

## Results

In this study, 200 women in both case and control groups were examined for the cervical levels of IL-6 and IL-8. The mean age was 44.39±12.58 years old in the case group and 43.46±12.35 years old in the control group (p=0.6). The mean marital duration was 23.48±12.3 years in the case group and 22.3±11.99 years in the control group, showing that the two groups had no significant difference (p=0.49). 

In this study, the cervical levels of IL-6 and IL-8 were evaluated in the two groups, including a healthy control group and a group of patients with premalignant lesions. The analysis by Mann-Whitney test showed that the difference in terms of the cervical levels of IL-6 and IL-8 was significant between the two groups. As shown in Table 1, the IL-6 level in the case group was more than twice the control group, and the IL-8 level in the case group was 30% higher than that of the control group.

The regression analysis showed that after the adjustment for age and marital duration, the case group had higher levels of IL-6 by 354.12 units with standard error of 63.23 (β=0.37, P<0.001) compared to that of the control group. Also, the cervical level of IL-8 after the modification for age and marital duration remained significant. The IL-8 level in the case group was higher by 263.76 units with standard error of 116.03 (β=0.16,P=0.02) compared to that of the control group (Table 2).

**Table 1:** Comparison of Mean Cervical Level of Interleukin 6 and 8 in the Studied Groups

Variable	Group	Mean (SD)	P-Value*
IL-6	Premalignant	568.66 (594.6)	<0.001
	Normal	212.7 (213.9)	
IL-8	Premalignant	1320.43 (876.5)	0.02
	Normal	1053.59 (747.6)	

*, Mann- Whitney U test

**Table 2.** Regression Analysis between the Two Groups in Terms of IL-8 and 6 Depending on Age and Marital Duration

Variable	Model	Non-standard coefficients		standard coefficients	t	P-value*
		B	Standard Error	β		
IL-6	Constant	629.44	269.34		2.34	0.02
	Group	354.12	63.23	0.37	5.6	<0.001
	Marital Duration	15.3	12.62	0.39	1.21	0.23
	Age	-17.44	12.30	0.45	-1.42	0.16
IL-8	Constant	1192.89	494.23		2.41	0.02
	Group	263.76	116.03	0.16	2.27	0.02
	Marital Duration	8.62	23.16	0.13	0.37	0.71
	Age	-7.63	22.57	-0.11	-0.34	0.74

*, Mann- Whitney U test

## Discussion

The present study was designed and implemented to compare the cervical levels of IL-6 and IL-8 in the patients with premalignant and malignant cervical lesions and normal women and also to explore its association with cervical premalignant lesions. The results of this study show that the cervical levels of these two cytokines were significantly higher in the patients with premalignant cervical lesions compared to the normal population. However, it was found that interleukins level had no correlation with patient age and marital duration. According to the literature review, limited studies have been conducted in this regard, so far. In a study performed by Tjiong et al., (1999) on 35 women with invasive cervical cancer, the mean cervical level of both interleukins in case group was significantly higher than that of healthy population. In another independent study, Tjiong et al., (1997) investigated the association between cervicovaginal IL-6 levels in women with cervical premalignant lesions and reported that the cervical IL-6 levels were significantly higher in patients group than that of control group. Moreover, Naik et al., (2016) in their research observed the increased levels of IL-6 in cervicovaginal secretions in patients with premalignant and malignant cervical lesions. Wei et al., (2001) also examined the cytosolic IL-6 level in malignant and non-malignant cervical tissue samples and found that the cytosolic level of this cytokine in malignant samples was significantly higher than that of non-malignant samples.

IL-8 is normally secreted by normal cells in very small amounts, whose production and secretion increases following the increased levels of other pro-inflammatory cytokines such as IL-1 and tumor necrosis factor (TNF), bacterial and viral products, and cellular stress (Kurai et al., 2018). Previous studies conducted on the association among serum, intracellular, and extracellular levels of this factor showed that the IL-8 level significantly increases in the presence of malignancies such as melanoma, breast carcinoma, ovarian malignancies, and pancreatic cancer (Green et al., 1996; Chen et al., 2014; Chia et al., 2014; Yung et al., 2018). In some studies, to compare the IL-8 level with the concentration around the lesion, the tumor surrounding IL-8 levels were found to be significantly higher than those of the bloodstream (Kotyza, 2012). According to available evidence, it was initially thought that the level of IL-8 would increase following the development of the tumor and as part of the tumor secretion factors. However, subsequent studies showed that this cytokine has a tumorigenic function. The IL-8, besides being a potent angiogenic agent, leads to the progression of malignancy through the stimulation of both proliferation and metastasis(Conroy et al., 2018; Jia et al., 2018).

Human Papilloma Virus is the main etiological factor in the development of premalignant lesions and invasive cervical cancer (Dai et al., 2018). Although no information exists on the effect of different subtypes of HPV on cervicovaginal IL-8 level, the evidence regarding the effect of this virus on changes in the IL- 8 levels in other tissues is noteworthy. Shiau et al., (2013) investigated the association between HPV 16 and the levels of metalloproteinases 2 and 9, as two angiogenic factors. Accordingly, their results showed that the HPV 16, either directly or indirectly by IL-17 could lead to a significant increase in the level of IL-8, thereby inducing the production of metalloproteinases. Zhang et al., (2014) confirmed the effect of HPV 16 on increasing the secretion of angiogenic factors, especially IL-8. Of note, the impact of low-risk HPV subtypes on the IL-8 expression is completely different from that of high-risk subtypes. In a study conducted by Akgül et al. on human primary keratinocytes, it was observed that HPV 5 and HPV 8 led to a downregulation of IL-8 secretion (Akgül et al., 2010). The IL-6 also has a similar mechanism to IL-8 in the pathophysiology of cervical cancer, with the difference that the IL-6 tumorigenic activity is often applied by stimulating proliferation and reducing apoptosis (Fisher et al., 2014). Information regarding the association of this cytokine with cervical cancer are greater than those of IL-8. Based on the results of some previous studies, IL-6 gene pleomorphism and its increased serum and local levels are associated with the incidence of premalignant and malignant cervical lesions (Shi et al., 2014; Liu et al., 2017).

An increase was observed in IL-6 secretion in studies conducted on cervical cells and other tissues infected with high-risk HPV subtypes. This increase in production and secretion, as in the case of IL-8, is made up of two direct and indirect routes, which were mediated by IL-17 (Hsiao et al., 2013; Ren et al., 2013). The serum or extracellular levels of IL-6 and IL-8 vary under various conditions. Hence, these two factors have been proposed as diagnostic markers for different pathologies. Moreover, the increased serum and extracellular levels of IL-8 and IL-6 have been reported in acute pyelonephritis, vesicoureteral reflux, pulmonary infections, osteomyelitis, and both hematologic and non-hematologic malignancies (Sheu et al., 2006; Mahmoud et al., 2010; Shahzad et al., 2010). Since a wide range of pathologies can alter the serum levels of these cytokines, their serum levels cannot be used for screening or diagnosing. However, extracellular and tumor surrounding levels of IL-6 and IL-8 can more specifically help in distinguishing between benign and malignant conditions (Shahzad et al., 2010; Zarogoulidis et al., 2014). Although examining the extracellular levels of cytokines, which represent the pretumoral level, is difficult in many pathologies, in the case of cervix, due to the ease of access to extracellular secretions, these two factors as two valuable diagnostic markers can help in a more accurate detection of malignant lesions. There is little evidence on the roles of IL-6 and IL-8 in the pathogenesis, diagnosis, prognosis, and the type of treatment for cervical malignancies, so further studies are needed in this regard. As well, it is recommended to perform studies on diagnostic value of cervical levels of IL-6 and IL-8 in CIN.

One of the limitations of this study was that the level of interleukins was not measured in terms of the CIN grade, so it is necessary to consider this issue in future studies.

In conclusion, this study showed that the cervical levels of IL-6 and IL-8 are associated with cervical intraepithelial lesions, independent of age and marital duration factors. It is suggested that future studies evaluate and compare the cervical levels of IL-6 and IL-8 in patients infected with different subtypes of HPV. Investigating the association of cervical levels of IL-6 and IL-8 with the treatment response rate as well as the survival rate of patients with cervical cancer, should also be done in future studies. 

## Author Contribution Statement

ZV: Supervision of the study and critical revision of manuscript. MA-K: Literature review, study design and manuscript writing. M S: Statistical analysis and its interpretation. A P: Perform Laboratory tests and critical revision of manuscript. M M: Data collection and manuscript writing.

## Source of funding

This study is a part of a doctoral thesis on obstetrics and gynecology, which is supported by deputy of research, Kashan University of Medical Sciences (Grant no: 96222). In addition, this study was approved by the Ethics Committee of Kashan University of Medical Sciences, Kashan, Iran (Ethical code:IR.kaums.mednt.rec.1396.116 date of Issue:2018.03.05).

## Conflict of Interest

The authors declare that they have no conflict of interest. 
